# Performance Evaluation of Deep Learning Models on Mammogram Classification Using Small Dataset

**DOI:** 10.3390/bioengineering9040161

**Published:** 2022-04-06

**Authors:** Adeyinka P. Adedigba, Steve A. Adeshina, Abiodun M. Aibinu

**Affiliations:** 1 Department of Mechatronics Engineering, Federal University of Technology, Minna 920211, Nigeria; abiodun.aibinu@futminna.edu.ng; 2 Department of Computer Engineering, Nile University of Nigeria, Abuja 900001, Nigeria; steve.adeshina@nileuniversity.edu.ng

**Keywords:** breast cancer, deep convolution neural network, discriminative fine-tuning, mammogram, mixed-precision training

## Abstract

Cancer is the second leading cause of death globally, and breast cancer (BC) is the second most reported cancer. Although the incidence rate is reducing in developed countries, the reverse is the case in low- and middle-income countries. Early detection has been found to contain cancer growth, prevent metastasis, ease treatment, and reduce mortality by 25%. The digital mammogram is one of the most common, cheapest, and most effective BC screening techniques capable of early detection of up to 90% BC incidence. However, the mammogram is one of the most difficult medical images to analyze. In this paper, we present a method of training a deep learning model for BC diagnosis. We developed a discriminative fine-tuning method which dynamically assigns different learning rates to each layer of the deep CNN. In addition, the model was trained using mixed-precision training to ease the computational demand of training deep learning models. Lastly, we present data augmentation methods for mammograms. The discriminative fine-tuning algorithm enables rapid convergence of the model loss; hence, the models were trained to attain their best performance within 50 epochs. Comparing the results, DenseNet achieved the highest accuracy of 0.998, while AlexNet obtained 0.988.

## 1. Introduction

Cancer is the second leading cause of death globally, accounting for about one in every six deaths reported worldwide. Breast cancer (BC) is the second most reported cancer, with about 2.09 million reported cases and 627,000 deaths in 2018 alone [1]. Although the incidence rate is reducing in developed countries, the reverse is the case in low- and middle-income countries; for instance, African countries accounted for 50% of the reported cases and 58% of deaths in 2018. Moreover, BC survival rate has increased to about 80% in North America (70% among black women on the continent) and 60% in Sweden and Japan, whereas it remains less than 40% in low-income countries [1]. This is because the low- and middle-income countries have inadequate health management facilities such as diagnosis and treatment facilities; this results in late detection and late-stage treatment among women with the disease [2].

Breast cancer is common among women, although a few cases among men have been reported [3]. BC is a malignant growth that starts from either the lobules or the milk duct of the breast. Ductal carcinoma in situ (DCIS) is a precancerous condition that begins its growth and is contained in the milk duct; it is considered the earliest appearance of BC and is easily detected by breast exam. Similarly, lobular carcinoma in situ (LCIS) is an abnormal growth that begins and is contained in the milk-producing lobule cells but does not invade or spread to other parts of the breast. However, unlike DCIS, LCIS is not easily detected by breast exam. BC can be invasive; a cancer that begins in the milk duct but spreads to other parts of the breast is called invasive ductal carcinoma (IDC), while one that grows from the lobule cells and then spreads to the other parts of the breast is called invasive lobular carcinoma (ILC). Lastly, BC can be metastatic when the cancer cells penetrate the circulatory or lymph system, spreading to other parts of the body via the bloodstream.

Early diagnosis of BC has been found to constrain cancer growth, prevent spreading, ease treatment, and reduce the mortality rate by 25% [4,5]. BC diagnosis techniques include breast exams, biopsy, mammograms, breast ultrasound, and magnetic resonance imaging (MRI). Digital mammographic screening is the most common, cheapest, and most effective BC screening technology capable of detecting up to 90% BC even before a lump can be felt by breast exam [6]. It uses a low-dose X-ray imaging of the breast where tissues in the breast, including tumors, appear as different shades of gray on the image. This makes mammogram screening the choice diagnostic technique in low- and middle-income countries.

**Figure 1:**
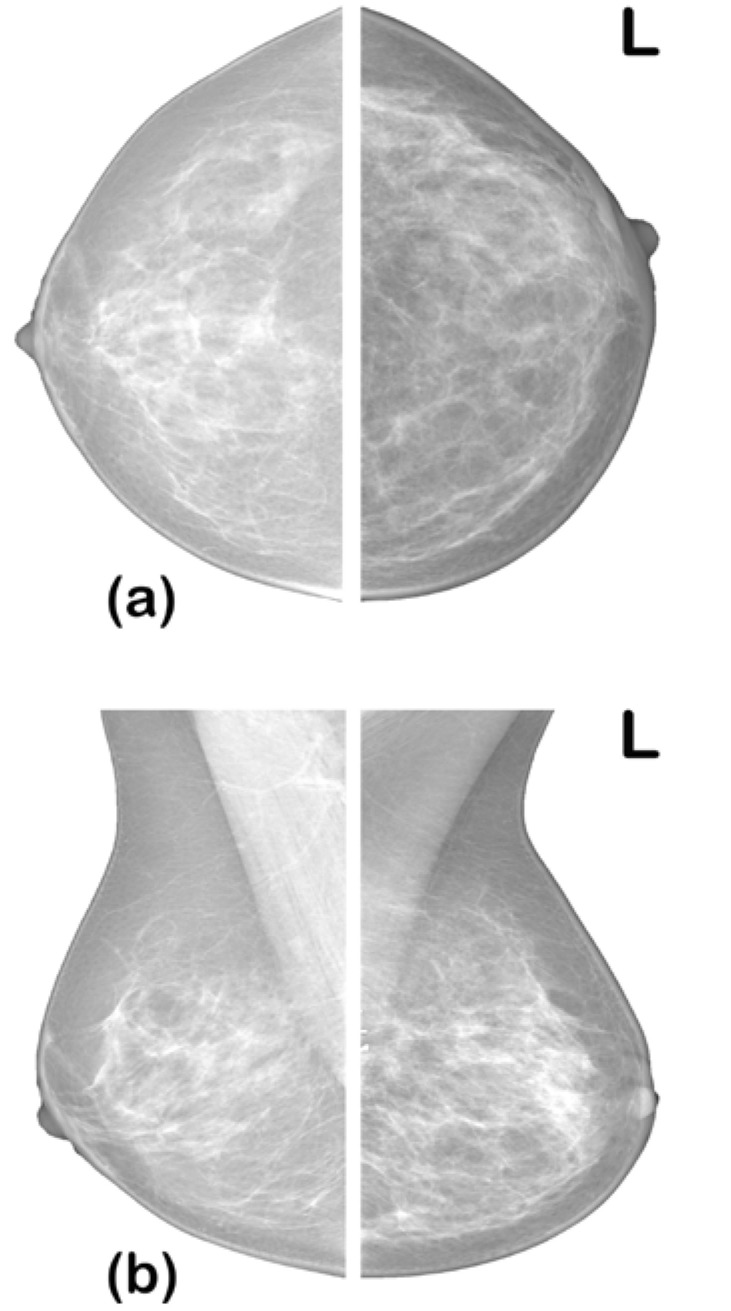
Mammogram views: (**a**) craniocaudal view; (**b**) mediolateral oblique view [7].

In diagnosing BC from a mammogram, radiologists look for specific abnormalities such as architectural distortion of breast tissue, alignment of the two breasts, masses, and calcification. Mammograms of the two breasts are taken from two views—the craniocaudal view (top-bottom view) and mediolateral oblique (MLO) view—to give the radiologist a comprehensive view for the examination (see Figure 1). Radiologists interpret their diagnosis using a standardized breast imaging reporting and data system (BI-RADS) scale developed by the American College of Radiology (ACR) [7]. The BI-RADS scale ranges from categories 0 to 6 detailed in Table 1.

However, due to low contrast, mammogram images are among the most difficult medical images to analyze. The sensitivity of mammograms is greatly affected by breast density and fats, which are radiolucent; hence, their appearance is similar to mass or calcification in the image [6]. As a result, the sensitivity of mammograms to early detection and accurate diagnosis has been estimated at 85–90% [8]. Today, medical centers face the challenge of screening an increasingly high volume of mammograms for accurate diagnosis, including early detection. To assist the radiologist, computer-aided diagnostic (CAD) systems have been proposed to reduce misdiagnosis. The developed CAD systems are based on different machine learning techniques [9,10,11]. However, the most successful of these techniques are based on deep convolution neural networks (CNNs) for the detection of mammograms [12,13]. While this method has produced very commendable results, it suffers from data availability. Deep CNN models require substantial training data (in order of hundreds of millions) to achieve high accuracy, sensitivity, and specificity; meanwhile, the medical image dataset is usually scanty (available in tens of thousands). In addition to data availability, deep CNN requires high computational power. This twin problem has greatly limited the clinical application of these models.

In this paper, we show that this twin problem can be addressed by a data- and computation-efficient method of fine-tuning deep learning models. We propose layer-wise discriminative fine-tuning and mixed-precision training, which both enhances high-speed convergence and improves accuracy. Lastly, we compare the performance of five top deep learning models trained on these techniques. Thus, in addition to implementing discriminative fine-tuning and mixed-precision training for super-convergence, this paper aims to demonstrate, by experiment, the best deep CNN models for mammogram classification, especially when data are scanty, to inform and guide future research and development properly.

The remainder of this paper is organized as follows: the theoretical framework and a review of related works are presented in Section 2; the dataset augmentation technique, discriminative fine-tuning, and mixed-precision training are presented in Section 3 along with the architecture of models employed in transfer learning; lastly, the results are presented in Section 4.

## 2. Review of Related Works

It has been shown that the generalization error of deep CNN increases substantially when the training example is small [14]; accordingly, all the state-of-the-art models were trained on a very large dataset (typically hundreds of millions of training data points) to ensure their training, validation, and test accuracy. However, medical images are available in a limited number (fewer than hundreds of thousands). Therefore, an effective algorithm needs to be developed to adapt a network trained on domains with voluminous training data to the small dataset available in the medical domain. Domain adaptation techniques are effective since they provide a mechanism of transferring knowledge from a source domain (for instance, domain with voluminous training examples) to a target domain (where training data are scarce) by exploring domain-invariant structures that underline distribution discrepancy in the two domains. Transfer learning, an example of a domain adaptation technique, is a method of retraining a previously trained deep CNN (base model) in a way that facilitates the reuse of its learned features and applying them to a new task (target model) by fine-tuning their fully connected layers only [15].

### 2.1. Methods of Transfer Learning

Transfer learning involves retraining a previously trained model (base model) on a new dataset from the current problem (target) domain. Depending on the similarity of the target domain and the domain where the base model is trained (usually called source domain), transfer learning can be feature extraction or fine-tuning. Feature extraction is usually applied when the target domain dataset is scanty and similar to the source domain. This is achieved by replacing the last fully connected layer of the base model architecture with a new layer corresponding to the target output, initializing the other layers with the weights from the previous training scenario, and retraining only the newly added layer. On the other hand, fine-tuning is applied either when the dataset is scanty or when the problem domains are different. This is achieved by replacing the last layer of the base model with a new layer corresponding to the target output, initializing the other layers with weights from the previous training scenario, and training the entire network again.

There are various methods of performing transfer learning to avoid overfitting the model, a few of which are as follows:

- **Self-tuning transfer learning** [16]: this method combines semi-supervised learning (SSL) with transfer learning. The SSL creates a pseudo-labeled dataset by exploring the latent structure of an unlabeled dataset which is then used to fine-tune the base model. The self-tuning transfer learning (STTL) algorithm enables a joint exploration of labeled and unlabeled datasets to create a larger dataset for transfer learning of a pretrained base model. However, since a model is as good as its labeled data, this method could introduce inaccurately labeled data into the dataset, which significantly limits its use in a medical scenario.
- **Adversarial fine-tuning** [17]: this method provides a fine-tuning technique for adversarial training (AT). AT introduces adversarial attacks into deep learning data, making the model robust to noise. However, training AT from scratch (just like any other deep learning method) incurs a high computational cost and, when using few data, could result in extreme overfitting. Adversarial fine-tuning (AFT) presents a transfer learning method in AT by optimizing the learning rate. Using a slow to fast learning rate scheduling during AT [17] demonstrates a significant reduction in computational cost and improved model accuracy. This method was applied to skin cancer detection in [18] to achieve an improved sensitivity of +5.67% but a slight improvement (+0.78%) in accuracy compared to other methods [18].
- **Intra fine-tuning** [19]: while transfer learning can be achieved regardless of the problem domain of the base model, the intra fine-tuning (IFT) method is applied in a non-distance dataset, i.e., intra-domain. Compared to a transfer learning from ImageNet, IFT showed a significant reduction in computational time but no improvement in training and validation accuracy [19].
- **Image-specific fine-tuning [20]**: this method provides image-specific adaptation to unseen object classes, i.e., zero-shot learning for image segmentation. Like STTL, this method also explores both supervised and unsupervised labeling approaches for image bounding boxes. Moreover, it uses a weighted loss function for interaction-based uncertainty in the fine-tuning process to limit the effect of the inaccurate label.
- **Learning to Reweight [21]**: this method uses meta-learning to reassign weights to the deep learning model on the basis of the direction of their gradient flow. A meta gradient descent step was performed on each mini-batch example to minimize the loss and validated on the validation set. The authors claimed that the method needs no additional hyperparameter tuning and is robust to class imbalance. Although this method has not been applied to computer vision, it reportedly achieved a boost improvement in natural language processing.

In summary, while traditional transfer learning can be computationally intensive, take a considerable time to converge, and is prone to overfitting, the above methods carefully avoid that. This shows that a careful fine-tuning of a deep learning model could improve generalization and allow faster convergence. However, the methods reviewed above are limited in scope, applicability, significant improvement, and generalization. In this work, we present discriminative fine-tuning, which dynamically assigns different learning rates and momentum to each layer of the network, unlike AFT which performs a learning rate schedule. DFT has a wide range of applications; it can be applied to different domains, image segmentation, language models, etc. It also works with any optimizer, as well as additional regularization techniques.

### 2.2. Mammogram Classification Using Transfer Learning

Many researchers have exploited these mechanisms to obtain good classification results from medical images, especially mammograms; some also compared the results of different base models. The detection of calcification and masses was presented in [22] using the feature extraction approach. Four state-of-the-art (SOTA) CNN models were trained as feature extractors by freezing all but the last layer of the models. In addition, instead of training on a full mammogram image, the models were trained on patches with the aim of localizing the abnormalities; a total of 2500 patches were used to train the models. After training, VGG achieved the highest overall accuracy of 92.53%, while AlexNet, GoogleNet, and ResNet achieved 91.23%, 91.10%, and 91.80%, respectively [22]. Although the author performed feature extraction, which trained only the last layer of the networks, the training demanded high computational power (a workstation with an NVIDIA GeForce TITAN X GPU was used) and longer compute time (up to 8 h for VGG).

Classification of mammograms into benign calcification, malignant calcification, benign mass, and malignant mass was presented in [23]. The ResNet50 model was fine-tuned in two stages. In the first stage, patches from the curated breast imaging subset of the digital database for screening mammography (CBIS-DDSM) were used to fine-tune the model. This fine-tuning was carried out using three-stage learning rate schedules: (1) learning rate was set to 10^−3^, and only the last layer was trained for three epochs, (2) learning rate was set to 10^−4^, and only the last 46 layers were trained for ten epochs, and (3) learning rate was set to 10^−5^, and all the layers were trained for 37 epochs. In the second stage, the whole mammogram was trained using two-stage schedules: (1) learning rate was set to 10^−4^, with a weight decay of 0.001, and only the last layer was trained for 30 epochs, and (2) learning rate was set to 10^−5^, with a weight decay to 0.01, and all layers were trained for 20 epochs. A mean accuracy of 99% was achieved at the 99th epoch of training the ResNet50 model. The model was trained on a workstation with an NVIDIA 8 GB Quadro M4000 GPU (indicating a high computation demand). However, it can be seen that the learning rate schedule strategy of fine-tuning paid off, as shown by good improvement within a few epochs of training compared to [22].

Classification of whole mammograms using the transfer learning approach was presented in [24]. The authors used mammograms acquired from the University of Kentucky Medical Center to fine-tune AlexNet and ResNet50 models. The models were trained using two groups of four NVIDIA 8 GB GTX 1080 GPUs (illustrating the high computational demand of the fine-tuning process). The model achieved the best AUC of 0.7274 with AlexNet [24]. Similarly, a performance comparison of the SOTA CNN model on the CBIS-DDSM dataset was presented in [25]. The study aimed to show the improvement of the transfer learning method over training from scratch. The author trained five SOTA models using a fixed learning rate. Although all models achieved an improved performance above training from scratch, the highest accuracy of 75.5% was obtained by ResNet152, and the best AUC of 0.804 was obtained by ResNet50 [25]. From these results, we note that not only does the traditional transfer learning approach demand high computational power, but it is also prone to overfitting, as shown by the AUC obtained.

A deep adversarial domain adaptation for breast cancer screening was presented in [26]. A model trained on CBIS-DDSM was adapted to new data acquired from West China Hospital. A two-stage approach was adopted. In the first stage, adversarial adaptation was performed where the input was CBIS-DDSM, which was used to train the adversarial network. By fixing the generator, the classifier was updated by maximizing the discrepancy ratio of the target domain; subsequently, the classifier was fixed so that the generator could be updated by minimizing the discrepancy on the target domain. In the second stage, the performance of the model on the target domain was improved by performing a case-level end-to-end training that extracted additional features and fused them with features learnt from the source domain. Although the target domain had a smaller dataset, the model achieved the highest accuracy of 85.15% with ResNet34 and the best AUC of 0.86 with ResNet101 [26]. This process was quite complex and demanded high computational power; the model was trained on a Tesla K40m 12 GB GPU for 400 epochs. In this paper, we show that better results can be achieved within a few epochs, conserving energy and demanding low compute power.

In [27], breast cancer classification from breast ultrasound using probability-based deep learning feature fusion was presented. A pretrained DarkNet53 model was used for feature extraction; these features were reduced using reformed differential evaluation and reform gray wolf algorithms. The best features were then fused using a probability-based serial approach. These features were then fed into different machine learning classifiers. The cubic support vector machine (C-SVM) classifier obtained the highest accuracy of 99.3% on the dataset.

Most of the papers reviewed used the CBIS-DDSM dataset; the work of [28] was validated on the INBreast dataset, which serves as a good comparison and benchmark for our method. In [28], feature extraction was performed using multifractal dimensions to extract multiple features from five different regions of interest blocks. The extracted features were reduced using a genetic algorithm; then, the reduced features were used to train the artificial neural network. The method was validated on four popular mammogram datasets, and the best result was obtained on the INBreast dataset. The highest accuracy of 99% (binary classification) was obtained, with sensitivity and specificity of 98.44 and 100%, respectively.

Table 2 shows a summary of the related works considered. From the table, most fine-tuning algorithms demand high computational power, as evident by powerful GPUs, with longer training episodes. The DFT presented in this work is capable of obtaining the best performance within a few epochs of training with low computational demand and low memory footprint, facilitated by mixed-precision training. In addition, most works focused on the binary classification of CBIS-DDS, which is not quite informative to the radiologist. In this work, we consider classifying mammograms using the BI-RAD numbers, which is a diagnostic and reporting standard among radiologists.

## 3. Materials and Methods

**Figure 2:**
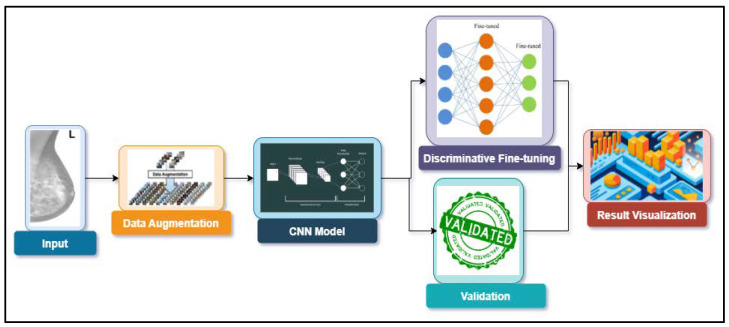
Block diagram of the proposed methodology.

The summary of this work is presented in the block diagram of Figure 2. The details of the block diagram are presented in this section. The dataset is discussed in Section 3.1, followed by data augmentation, while the SOTA CNN models used are discussed in Section 3.3. The discriminative fine-tuning technique and mixed-precision training are discussed next, giving the complete training process. Validation and a discussion of the results are presented in the next section.

### 3.1. Dataset

**Figure 3:**
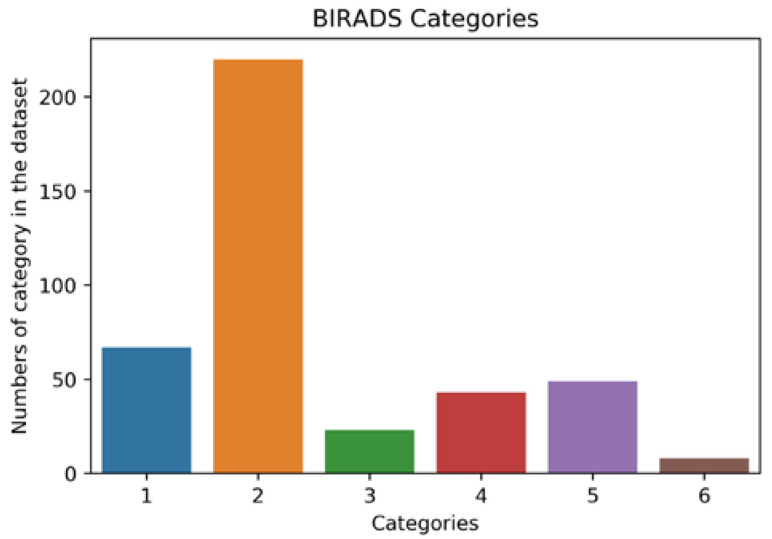
Distribution of BI-RADS categories in the dataset.

To demonstrate how a small dataset can be utilized to achieve great training and validation accuracy, the INBreast dataset was used [7]. The dataset was gathered at the Breast Center, Porto, with permission from the Hospital’s Ethics Committee and National Committee of Data Protection. The dataset consists of 117 cases, out of which 90 cases have two views (CC and MLO views) of the breast pair, and 25 cases underwent mastectomy leaving only one breast for the two views. The total number of images in the dataset is 410. The images are available in the standardized digital imaging and communication in medicine (DICOM) format, in which medical images are stored with the patient’s details and medical history. However, all confidential information was removed in line with data protection protocol. The nonconfidential information available was the breast description and BI-RADS category, as summarized in Figure 3.

### 3.2. Data Augmentation

In addition to being a small dataset, the INBreast dataset is highly biased toward a particular breast finding and BI-RADS category (see Figure 3); this is usually referred to as a class imbalance problem. The category or class with a higher number in the dataset is called the majority group, whereas the that in a smaller number is called the minority group. Class imbalance presents a challenge to the deep learning model; it results in over-classification of the majority group, while the minority class is misclassified as belonging to the majority group.

Techniques for handling class imbalance include random minority oversampling, random majority undersampling, and cost-sensitive learning [29]. Cost-sensitive learning presents additional computational cost to the training algorithm, whereas random majority undersampling leads to the loss of valuable data. Hence, random minority oversampling was employed in this work to even out the imbalance.

On the other hand, training a deep convolution network with a small training dataset leads to overfitting. To prevent this, data augmentation is very imperative. Augmentation in this project was conducted with two goals: increasing the dataset and increasing variance within the dataset. We noted that the mammographic image quality was affected by contrast, sharpness, exposure, and noise, all of which depended on the machine. Therefore, we simulated these factors and synthesized additional images to augment our scanty dataset.

The additional images were synthesized by randomly performing Gaussian blurring, intensity shifting, internal rotation, and mild white noise.

Gaussian blurring applies two-dimensional Gaussian filters on the input image to remove noise; however, in this case, Gaussian blurring was used to add within-class variance to the dataset. The filter is developed as an extension of one-dimensional Gaussian filter, given by

(1) $$G\left(x\right)=\frac{1}{\sqrt{2\pi \sigma^{2}}}e^{-\frac{x^{2}}{2\sigma^{2}}}$$

Thus, the two-dimensional Gaussian filter is given by

(2) $$G\left(x,y\right)=\frac{1}{\sqrt{2\pi \sigma^{2}}}e^{-\frac{x^{2}+y^{2}}{2\sigma^{2}}}$$

where *σ*^2^ is variance of the Gaussian filter. It can be noticed that Equation (2) is a product of two Gaussian filters. Applying Equation (2) as an image filter to pixel coordinate (*r,c*) according to [30] yields

(3) $$G\left(r,c\right)=e^{\frac{{\left|\left|r-c\right|\right|}^{2}}{v}}$$

where *r* is the row, and *c* is the column coordinate. From Equation (3), Gaussian blurring works by adjusting the Euclidean distance between neighboring pixel intensities. Blurring is a common phenomenon encountered in medical images. It is usually introduced during the process of capturing the mammographic image. Thus, introducing Gaussian blurring to the training dataset increases the dataset and increases the variance within the dataset, making the model more robust.

The image intensity shifting was simulated to reflect poor mammogram exposure, which could affect the performance of both the radiologist and our algorithm. Gamma intensity transformation was employed here; it is mathematically given by

*I*^0^ = *cI^γ^*, (4)

where *c* and *γ* are nonzero constant values, and *I*^0^ is the new intensity shifted by *c* and *γ*.

Likewise, image rotation is achieved by randomly adding or subtracting a small angle *φ* from the coordinate of the original image; this is mathematically given by

(*r*^0^,*c*^0^) = *rcosφ* ± *csinφ*. (5)

The overall augmentation algorithm was as follows: an image was randomly picked (with replacement) from the dataset. The three augmentation transformations to be performed were defined, and then a pipeline of these augmentation transformations was formed, which was then randomly selected from and applied to the image. After the transformation was applied to the image, the new augmented image was then saved to disk. Pseudocode 1 for this algorithm is summarized below.
**Pseudocode 1: Data augmentation algorithm.**
**Input: **(D: Dataset, T: list of augmentation transformations)
**Output:**$D_{aug}:$ Augmented Dataset**Step 1:**Randomly select an image ℐ from D
**Step 2: **Randomly select an augmentation transformation ℱ from T
**Step 3:**Randomly select the parameter 𝒫 for ℱ
**Step 4:**Apply the augmentation transformation ℱ on the image ℐ using the parameter 𝒫**Step 5:**Save the image ℐ in the augmented dataset $D_{aug}$


### 3.3. CNN Model Architecture

To test the hypothesis, transfer learning was performed using popular pretrained CNN models. In this project, AlexNet, VGG, ResNet, DenseNet, and SqueezeNet were used; the architectures, as well as the central design idea of each of these networks, are discussed below.

**AlexNet [31]**: this model is an eight-layer network consisting of five convolutional layers and three fully connected layers, pretrained on the high-resolution ImageNet dataset. AlexNet, developed by Alex Krizhevsky, Geoffrey Hinton, and Ilya Sutskever, won the 2012 ImageNet competition with a 15.3% top five error rate and has since become one of the baseline models in CNN history.

**VGG [32]**: this model is a 16-layer CNN developed by the Visual Geometry Group, Oxford University. The model was pretrained on the ImageNet dataset for the ImageNet competition. VGG was the first runner-up of the 2014 ImageNet classification task. VGG is desired for its uniform 3 × 3 convolution kernel used in building the model’s architecture; due to its simple kernel structure, it has become a favorable model for feature extraction tasks.

**SqueezeNet [33]**: this model achieves similar performance to AlexNet but with 50% fewer parameters. The parameter reduction was achieved using 1 × 1 filter instead of larger filters and decreasing the number of input channels to their 3 × 3 filters. This follows from [32,34] where smaller filters were shown as an approximation of larger filters. Thus, instead of using larger filters, smaller filters are repeatedly used throughout the network, guaranteeing parameter reduction. On the other hand, the accuracy is maintained by ensuring that each convolution layer receives large activation maps from the previous layer; that is, pooling (or downsampling) is not applied to earlier layers of the network. These key intuitions are implemented in the *fire* module of the network, which comprises a *squeeze* module (1 × 1 filter) and an *expand* module (which has a 1 × 1 filter followed by 3 × 3 filter). The model was trained using similar parameters to AlexNet, and its performance was benchmarked against AlexNet. It was found that SqueezeNet performs as well as AlexNet, despite its fewer parameters [34].

**ResNet [35]**: generally, deeper convolutional networks outperform their shallow counterparts [36]; however, training a deeper model increases the training error rate due to the vanishing gradient problem. To solve this, ResNet introduced the residual block (Equation (6)), which creates a connection between the output of a convolutional layer and the original input to the layer using identity mapping [35]. Thus, the activation of a residual block is given as

**a***_l_* = U(**a***_l_* − _1_) + **a***_l_*_− 1_, (6)

where **a***_l_* is the activation of layer *l*, U(·) is a nonlinear convolutional transformation of the layer, and **a***_l_*_− 1_ is the activation of previous layer *l* − 1. The skip connection of Equation (6) enables more layers to be stacked on each other, resulting in a deep network. ResNet152, a 152-layer convolutional network, won the 2015 ImageNet competition with a 3.57% top five error rate, higher than human-level performance. In this work, ResNet101, a 100-layer convolutional network pretrained on the ImageNet dataset, was used.

**DenseNet [37]**: it is possible to train a much deeper network with fewer parameters and better accuracy than ResNet, by implementing a dense block (Equation (7)) instead of a residual block (Equation (6)). The dense block creates a form of connection that allows any layer within the network to be connected to all layers that follows it [32]. That is, layer *l* receives feature activations from all its preceding *l* − 1 layers as follows:

**a***_l_* = T([**a_0_**, **a_1_**, …, **a**_(***l***− **1**)_]), (7)

where **a** is the activation of the *l*-th layer, [**a**_0_, **a**_1_, …, **a**_(*l* − 1)_] is a concatenation of all the previous layer activations which can be seen as a form of collective information gathered by the network up to layer *l* − 1. T(·) is a nonlinear transformation that maps the concatenated activation to the activation of layer *l*. Comparing Equations (6) and (7), the element-wise operation of the skipped connection in Equation (6) results in a parameter increase in *O(C* × *C)*, whereas Equation (7) results in fewer parameters of *O(l* × *k* × *k),* where *C* is the number of channels, *k* is the growth order of the dense connection, and *l* is the number of layers. For example, ResNet101, a 101-layer convolutional network, has 10.2 M parameters, while DenseNet-BC (with *k* = 12), a 100-layer convolutional network, has 0.8 M parameters [37].

### 3.4. Discriminative Fine-Tuning and Mixed-Precision Training

To obtain a good generalization, deep learning models must be trained on a large, well-labeled training dataset, using a high-specification computer with graphics processing units for a very long time. Hence, the state-of-the-art models in computer vision were trained on ImageNet Large Scale Visual Recognition Challenge (ILSVRC) data, consisting of hundreds of millions of well-labeled training data. When such huge training data are not available or computational power is limited, the usual practice is to perform transfer learning (see Section 2).

Because fine-tuning involves training the entire network all over again, the performance of the model on the current problem depends on how well the training is conducted, in addition to the high demand for computational power, as shown in Section 2.2. Overfitting is one of the primary reasons for the poor performance of fine-tuned models. Overfitting occurs when a model performs very well on the training set but poorly on the test and validation set; such model performs woefully when deployed and should be avoided, especially in medical applications. Methods of overcoming overfitting include training with an extensive training set, data augmentation, and regularization. Regularization refers to techniques that make slight modifications to the learning algorithm such that the model generalizes better on the unseen dataset. Regularization can be achieved by optimizing hyperparameters such as learning rates, weight decay, batch size, and dropout.

In our previous work [29], we introduced discriminative fine-tuning, where we assigned different learning rates and momentum to each layer of the network. The idea is that we found that each layer of the network is learning different features and, as such, has different objectives. Hence, it would be good to tune each layer with different learning rates and momentum to facilitate the learning process without getting stuck in a local minimum or saddle point.

Mathematically, the parameter update scheme of the stochastic gradient descent (SDG) is given as

(8) $$\theta_{t+1}=\theta_{t}-\alpha \frac{dJ\left(\theta_{t}\right)}{d\theta_{t}},$$

where $\theta_{t}\;\mathrm{and}\;\theta_{t+1}$ are the network parameters in the previous iteration t and new iteration t+1; the parameters are adjusted by the gradient of the network objective $J\left(\theta_{t}\right)$, scaled by the learning rate α. The network objective is a function of network loss and the weight penalty (regularizer) given as

(9) $$J\left(\theta \right)=\frac{1}{D}\sum_{i=1}^{D}ℒ\left(ℱ\left(x_{i},\theta \right),y_{i}\right)+\lambda \Omega \left(\theta \right),$$

where ℒ(⋅) is the loss function, ℱ(⋅) represents the network output with a target or training label represented by $y_{i}$, and Ω(⋅) represents the regularization function scaled with λ.

By modifying Equation (8) such that each layer of the network parameters is fine-tuned with different learning rates, the network would converge more rapidly.

(10) $$\theta_{t+1}^{l}=\theta_{t}^{l}-\alpha^{l}\frac{dJ\left(\theta_{t}^{l}\right)}{d\theta_{t}^{l}}.$$

By fine-tuning each layer at a different learning rate, we facilitate each network layer to focus on learning its separate objective. This follows Zeiler and Fergus, who showed that each layer of the deep CNN learns different features, with earlier layers learning primitive features while later layers learn complex features.

#### Discriminative Learning Rate

How then do we select the learning rate for each network layer? The general wisdom is to select a smaller learning rate for earlier layers. Since a pretrained network has already learned primitive features, it is required to learn and select a larger learning rate for later layers for faster convergence.

In this work, the selection of optimal learning rates was applied experimentally. We designed a one-epoch training experiment where we varied the learning rate of the model for each iteration in the epoch and observed the resulting network loss. Then the value of the loss was plotted against the learning rate. The learning rates in the range of the steepest slope (highest derivative) of loss were selected. These learning rates were then distributed within the network layers, with the lowest value assigned to the earliest layer and the highest value assigned to the latest layer. The layers in between were assigned values according to a triangular law that increased the learning rate.

(11) $$\alpha^{l}=\{\begin{array}{c} \alpha_{min}+\frac{\alpha_{max}-\alpha_{min}}{\alpha_{max}}l\;if\;\kappa l_{max}\geq l<l_{max}\\ \alpha_{max}-\frac{\alpha_{max}-\alpha_{min}}{\alpha_{max}}l\;if\;\kappa l_{max}\leq l<l_{max} \end{array},$$

where $\alpha_{max}$ and $\alpha_{min}$ are the upper and lower bounds of the learning rate range determined from the experiment, l is the layer, and κ is a random number used to linearly vary the layers from lowest to highest layer. This idea was found to produce a better result and a good generalization [38,39]. The complete Algorithm 1 is listed below.
**Algorithm 1:** Discriminative fine-tuning algorithm.1:**Procedure** DFT2:
   **Input**: $(\alpha_{min}:$ minimum learning rate, $\alpha_{max}$: maximum learning rate, $m_{min}$: minimum momentum, $m_{max}$: maximum momentum, D: size of dataset, batch_size)3:
   **Output:**
(θ: Network parameters)4:
   $t\;\leftarrow \frac{D}{batch\_size}$
5:
   
κ←rand(0,1)
 //κ
*    //κ determines how rapidly the learning rate increase or reduces*


   
**while**
$\kappa t<\frac{1}{2}t_{max}$
6:

   **for**
t in each iteration **do:**7:


    **for**
l in each layer **do:**8:



   $\alpha_{t}^{l}\leftarrow \alpha_{min}+\left(\frac{\alpha_{max}-\alpha_{min}}{\alpha_{max}}\right)l$     //increase learning rate per layer9:



   $m_{t}^{l}\leftarrow m_{min}+\left(\frac{m_{max-m_{min}}}{m_{max}}\right)l$   //increasing the momentum per layer10:



   
${𝓋}_{t+1}^{l}\leftarrow m_{t}^{l}𝓋{\;}_{t}^{l}-\alpha_{t}^{l}\frac{dJ\left(\theta_{t}^{l}\right)}{d\theta_{t}^{l}}$
11:



   $\theta_{t+1}^{l}\leftarrow \theta_{t}^{l}+{\;}_{t+1}^{l}$             //update the layer parameters12:



   end **for**13.


   end **for**14.

   end **while**
15.
   **while**
$\frac{1}{2}t\leq \kappa t<t_{max}$
16.

   **for** t in each iteration **do:**
17.


   **for**
l in each layer **do:**
18.



   $\alpha_{t}^{l}\leftarrow \alpha_{min}-\left(\frac{\alpha_{max}-\alpha_{min}}{\alpha_{max}}\right)l$    //increase learning rate per layer19.



   $m_{t}^{l}\leftarrow m_{min}-\left(\frac{m_{max-m_{min}}}{m_{max}}\right)l$    //increasing the momentum per layer20.



   
${𝓋}_{t+1}^{l}\leftarrow m_{t}^{l}𝓋{\;}_{t}^{l}-\alpha_{t}^{l}\frac{dJ\left(\theta_{t}^{l}\right)}{d\theta_{t}^{l}}$
21.



   $\theta_{t+1}^{l}\leftarrow \theta_{t}^{l}+{\;}_{t+1}^{l}$          //update the layer parameters22.



   end **for**23.


   end **for**24.

   end **while**25.end **Procedure**

### 3.5. Experiment Setup

This experiment aimed to show the performance of different network architectures when trained with a small dataset following our proposed data augmentation technique and DFT. To achieve this, we trained five large CNN architectures using the transfer learning approach. As a result of the limited computation resources, some constraints were fixed while training. The constraints include (1) a reduction in the number of epochs ≤50, (2) a reduction in training time, and (3) Optimal performance of the model before the end of training time These constraints were achieved during training by adopting the DFT and mixed-precision training [40].

To implement DFT, a single-epoch trial experiment was first carried out. In the experiment, the learning rate was gradually increased for each new iteration, and the loss of the model was observed. The range of learning rate where the gradient of the loss was high was then taken as the learning rate for the discriminative fine-tuning. The result of this experiment is discussed in Section 4.

In addition to DFT, we implemented mixed-precision training to account for the training deep learning model’s huge computation and memory demand. The following variables usually demand large memory storage during training: weights, activation, input image, and output. These variables are usually stored using 32 bit IEEE single-precision floating point (FP32) numbers with 32 bit memory for every number in the tensor. Using FP32 results in high-precision operations at the cost of memory. By contrast, half-precision stored numbers using only 16 bit (FP16) requires less memory than FP32. Unlike the single-precision format, which is the de facto approach for training a large neural network, half precision has been shown to lead to faster training, achieving 2–8 times improvement while achieving comparable results [40]. Hence, in this work, mixed-precision training was used to accelerate training time without losing network performance.

Lastly, for the actual training of the deep CNNs, images in the dataset were first converted from DICOM format to portable graphic network (PNG) format, and then they were resized to 400 × 400 from their original resolution to reduce memory implications. Then augmentation algorithm presented in Pseudocode 1was implemented on these resized images; a total of 18,200 images were saved to disk after careful visual inspection. The dataset was cleansed of repeated images, images that did not preserve breast anatomy chirality, and so on. Finally, the new dataset contained 11,234 images. Then, the new dataset was divided into training, validation, and testing subsets using a ratio of 80:15:5. Mixed-precision training reduced the memory demand; hence, we increased the batch size to 64, which accelerated the training and validation. The setup was implemented on PyTorch and trained on an Asus laptop with an NVIDIA RTX 2070, Intel Core i7-8750H, and 16 GB of RAM.

## 4. Results

The experimental results are presented in this section. With the experimental setup discussed earlier, the state-of-the-art performance was obtained in just 50 epochs of training, with each epoch running for an average of 90 s. This epoch runtime resulted from mixed-precision training, which yielded a reduction in memory usage, allowing us to use a larger batch size than if the training was conducted in FP32 precision; this eventually accelerated the epoch runtime and training time. Likewise, the discriminative fine-tuning process allowed us to dynamically assign a learning rate to each layer of the model, resulting in quick convergence and high accuracy without overfitting.

**Figure 4:**
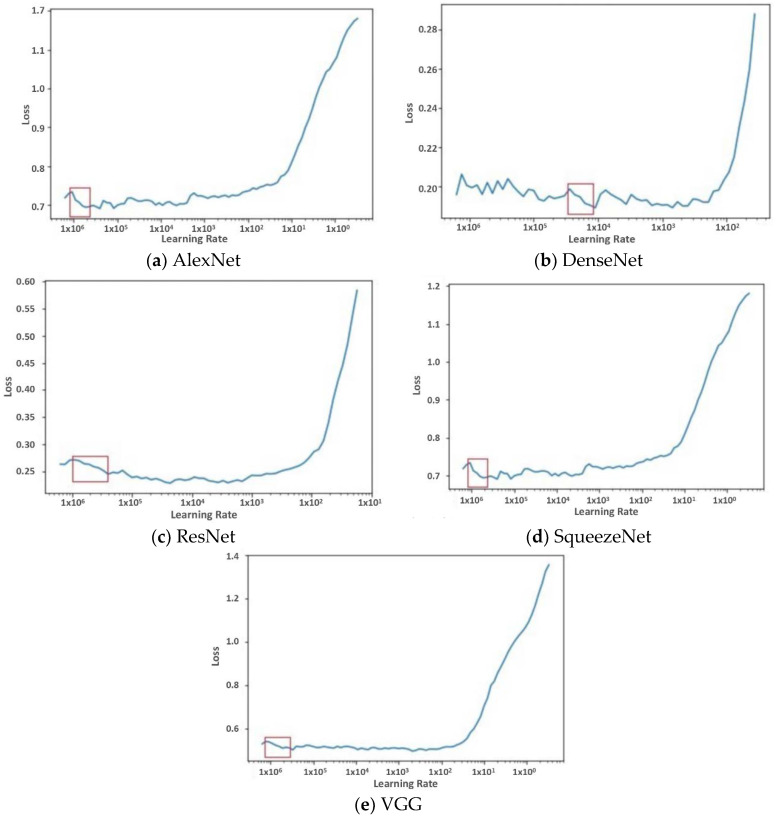
Finding the optimum learning rate that best optimized the loss function. The graphs show the variation of training loss with learning rate.

The choice of the learning rate for the DFT algorithm was accomplished by running a single-epoch trial experiment using different learning rates and observing how the loss function increased or decreased during this epoch. The single-epoch trial experiment was carried out for each model to guide our selection of the optimal learning rate used to train each model. The learning rate selected was within the range where the slope of the loss function reduced sharply (showing a high gradient). Figure 4 presents the result of this trial experiment obtained for each model; the red bounding box represents the learning rate selected for the model.

**Figure 5:**
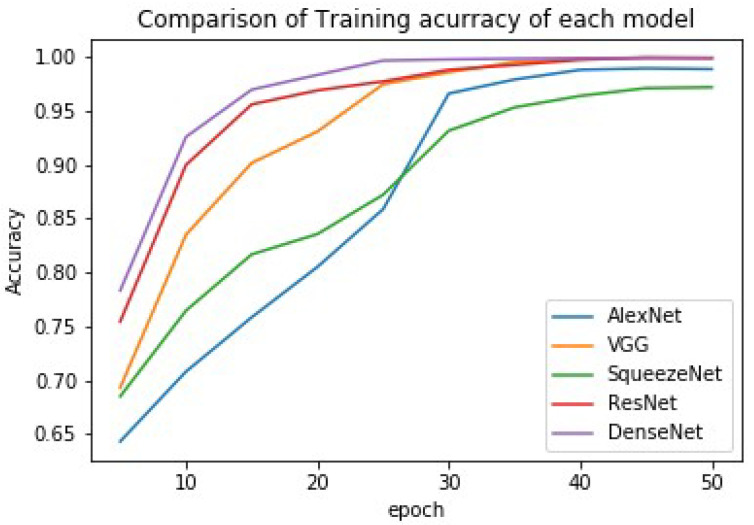
Comparison of training accuracy using different models.

The training was achieved using the DFT approach (see Algorithm 1), and the results of each model (the accuracy, precision, and recall) are presented in Table 3. A comparison of each model’s accuracy is graphically presented in Figure 5.

**Figure 6:**
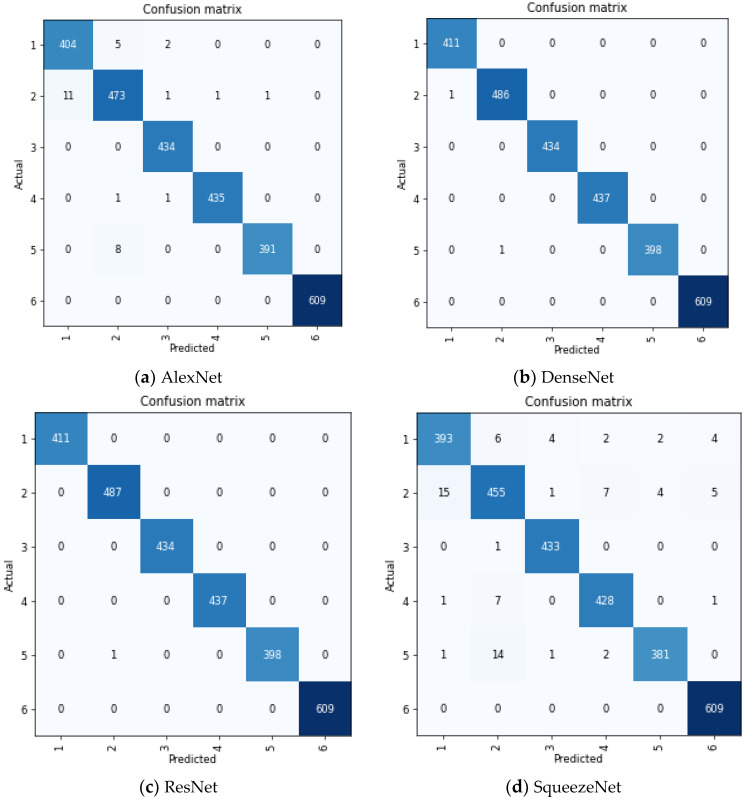

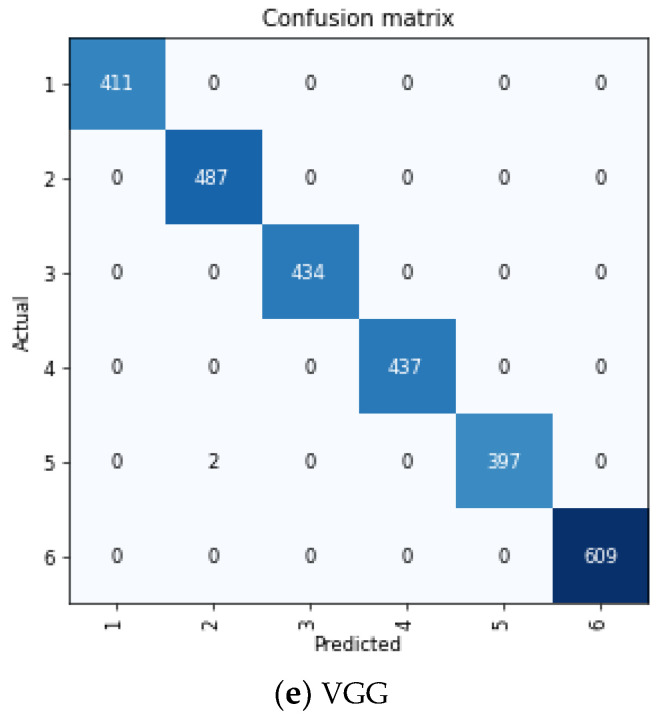
Confusion matrix of each model. The figure shows how accurate each model classified the classes in the dataset.

The confusion matrix for each model is presented in Figure 6. We adopted the multiclass sensitivity and specificity criteria in interpreting this confusion matrix. In multiclass classification, the sensitivity of a model is its ability to predict a particular class correctly. In contrast, sensitivity is the ability of the model to correctly predict that an image does not belong to a particular class [41]. This is mathematically given as

(12) $$MSN_{i}=\frac{TP_{i}}{TP_{i}+FN_{i}},$$

(13) $$MSP_{i}=\frac{TN_{i}}{TN_{i}+FP_{i}},$$

where

(14) $$TP_{i}={C_{ij}|}_{i=j},\;FP_{i}=\underset{i}{\sum }C_{ij}-TP_{i},\;FN_{i}=\underset{j}{\sum }C_{ij}-TP_{i},\;TN_{i}=\underset{i}{\sum }\underset{j}{\sum }C_{ij}-TP_{i}-FN_{i}-FP_{i},$$

where $TP_{i}$ is the true positive rate for class i which measures the number of images correctly classified as class i, whereas $TN_{i}$ is the true negative rate for class i which measures the number of images that is rightly classified as non-member of class i. Conversely, $FP_{i}$ is the false positive rate of class i which quantifies the total number of images that are wrongly predicted to belong to class i, whereas $FN_{i}$ is the false negative rate which gives the total number of images that belong to class i but the model predicted to belong to another class.

## 5. Discussion of Results

The Breast Imaging Reporting and Data System (BI-RADS) lexicon is a comprehensive, standardized breast imaging report developed by the American College of Radiology and widely adopted by radiologists. Hence, we think a good computer-aided diagnosis system should report a mammogram classification using the BI-RADS standard. However, many research works in developing a CAD system for breast cancer diagnosis are limited to binary classification (benign or malignant) partly because of the small dataset and the resulting curse of dimensionality.

**Figure 7:**
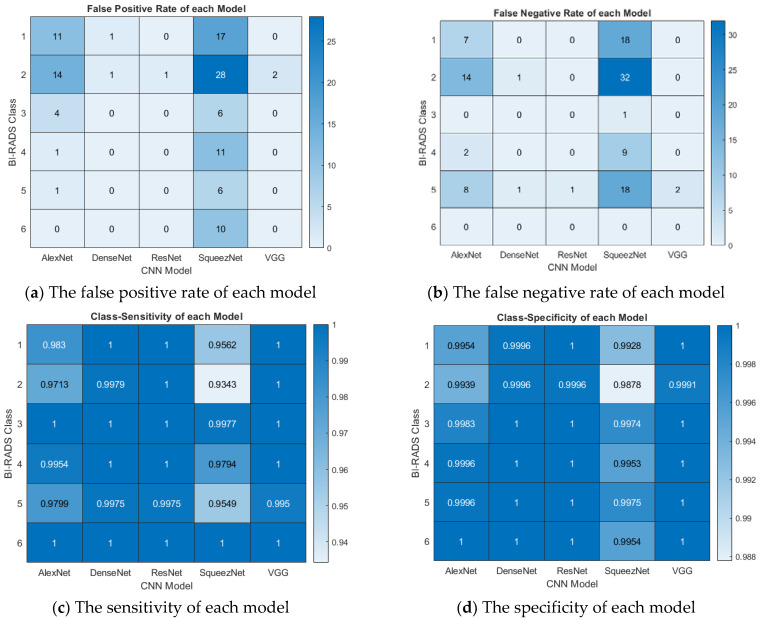
Interpretation of the results. The figure shows the false positive rate, false negative rate, sensitivity, and specificity of each model per BI-RADS category.

This paper developed a fine-tuning algorithm that works well with SOTA CNN models for multiclass classification using the BI-RADS numbers. Our algorithm achieved good classification accuracy and high precision and performed well with other metrics, as shown in Figure 7. The plots were generated from the confusion matrices shown in Figure 7 with formulae presented in Equations (12)–(14). On each plot, the CNN models are on the *x*-axis, while the BI-RADS class is on the *y*-axis.

The numbers of false positives (FPs) and false negatives (FNs) are presented in Figure 7a,b, respectively, showing the misclassification of the model output for each BI-RADS class. The FPs and FNs are of great interest in a medical diagnosis, especially cancer classification. The FP shows the number of cancers that can be reported as noncancerous, resulting in late treatment and eventual death of the patient. Conversely, the FN is the number of noncancerous (mild) cases falsely reported as cancerous; this could result in overdiagnosis, mistreatment, and physiological stress on the patient. Hence, FPs and FNs on an excellent CAD system should be minimal. As shown in Figure 7a,b, AlexNet and SqueezeNet presented high FPs and FNs. Furthermore, the figure shows that BI-RADS classes 2 and 5 were misdiagnosed because fatty breasts were sometimes misclassified as cancerous. Lastly, class 6 showed no sign of FNs in all models, while all models but SqueezeNet also showed no sign of FNs for class 6. This is because class 6 is known cancer with a well-defined tumor that can hardly be misdiagnosed.

Model sensitivity measures how often a model correctly identifies the cancer classes. This can be used to forecast how well the model will behave when deployed. The sensitivity of each model to the BI-RADS classes is presented in Figure 7c. The model’s specificity shows the probability of recognizing an image that does not belong to a particular class. The class specificity is shown in Figure 7d; the figures represent sensitivity and specificity as a probability (0 to 1). The figures show that ResNet and VGG were highly sensitive with high specificity. Furthermore, note that all models were highly sensitive to class 6 but showed a relatively weak sensitivity to classes 2 and 5, for the same reason provided above. Although classes 2 and 5 may be confused, our model showed high specificity, with a confidence factor <0.98.

**Figure 8:**
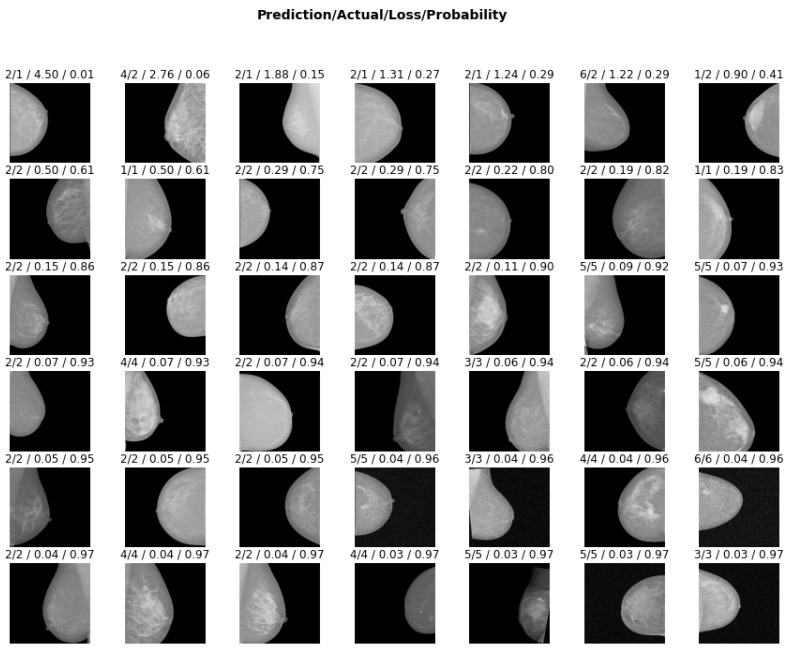
Visualization of ResNet prediction on 42 randomly selected test images. The following are shown on each image: the predicted class by the model, the actual class it belonged to, the loss for wrongly classifying the image, and the model’s prediction probability of the actual class (i.e., the probability when the output is the actual class). It should be noted that the images are arranged in decreasing order of model loss.

In addition, to show that DFT could prevent overfitting, we visualized the inference of the ResNet model on 42 randomly selected test images. A model which confidently predicts a wrong class shows signs of overfitting [29]. Hence, in Figure 8, the model was tested for overfitting by displaying each test image with the loss accrued by its decision and the prediction probability, i.e., confidence level of its decision. The figure shows that the majority of images were classified correctly with lower loss and high confidence (probability), while the wrong classes were established with a lower confidence level. Training with DFT enables each layer of the network to discover salient features that enhance its decision; therefore, misclassification is achieved with lower probability and high loss.

Although the INBreast dataset has received little attention in the literature, and fewer studies have been conducted on multiclass classification tasks, we compared our work with similar studies on the INBreast dataset reported in the literature, as shown in Table 4. Note that BI-RADS multiclass classification was presented in [42] with a poor result of 83.9% compared to 99.8% achieved in this work.

## 6. Conclusions

The performance comparison of deep learning models on the INBreast mammogram dataset was presented in this paper. The models were fine-tuned using the discriminative fine-tuning method, which dynamically assigns different learning rates and momentum to different network layers to achieve rapid convergence and high performance. Moreover, a multiclass classification based on the BI-RADS lexicon on the INBreast dataset was carried out. The results showed good accuracy with a low false positive rate, low false negative rate, high specificity, and high sensitivity of the models for each category of the BI-RADS classes. Compared with the literature, the models presented here improve the state-of-the-art results (to the best of our knowledge). Hence, discriminative fine-tuning works well with state-of-the-art CNN models, achieving excellent performance without overfitting on small datasets.

## Figures and Tables

**Table 1:** Description of BI-RADS categories.

Category	Diagnosis	Description
0	No findings	The mammogram does not provide sufficient information or findings are inconclusive. Follow-up examination may be recommended.
1	Negative	No BC traces or findings, although routine screening is recommended.
2	Benign	Confirmation of benign finding; routine screening is recommended.
3	Probably benign	Findings that have high probability (>0.98) of being benign; 6 month interval follow-up is recommended.
4	Suspicious abnormality	Probable (0.3–0.94) malignant growth; a biopsy is recommended.
5	Highly suspicious of malignancy	Abnormal growth that has high probability (≥0.95) of being malignant; doctor’s decision should be sought.
6	Proven cancer	Biopsy-confirmed malignant growth.

**Table 2:** Summary of related works.

Reference	Method	Dataset	Limitations
[22], 2018	Traditional feature extraction	CBIS-DDSM	High computational demand and long training episode (8 h)
[23], 2019	Gradual fine-tuning with episodes of learning rate annealing schedules	CBIS-DDSM	High computational demand and long training episode (99 epochs)
[24], 2018	Traditional fine-tuning	CBIS-DDSM	High computational demand, low AUC, and overfitting
[25], 2019	Traditional fine-tuning	CBIS-DDSM	High computational demand, low AUC, and overfitting
[26], 2021	Deep adversarial domain adaptation	CBIS-DDSM	Complex algorithm with high computational demand and long training episode (400 epochs)
[27], 2022	Feature extraction plus feature selection using twin algorithms: reformed differential evaluation and reformed gray wolf algorithm.	Breast ultrasound images	Long training episodes and additional computation burden introduced by feature selection algorithms
[28], 2021	Multifractal dimension feature extraction, feature reduction using GA, and classification using ANN	DDSMMini-MIASINBreastbreast cancer digital repository	Not end-to-end trained; each algorithm introduced computational bottlenecks that aggregated to high computational demandNot compatible with SOTA CNN models

**Table 3:** Training results of each model.

Model Name	Accuracy (%)	Precision (%)	Recall (%)
AlexNet	98.88	98.84	98.82
SqueezeNet	97.19	97.16	97.04
VGG	99.28	99.3	99.15
ResNet	99.5	99.7	99.5
DenseNet	99.8	99.82	99.77

**Table 4:** Comparison of our results with those reported in literature.

Reference	Dataset	Highest Accuracy Reported (%)
[28]	INBreast	99.0
[43]	INBreast	90.0
[42]	INBreast	83.9
[44]	INBreast	97.27
Our method	INBreast	99.80

## Data Availability

Publicly available datasets were analyzed in this study. This data can be found here: https://www.kaggle.com/martholi/inbreast (accessed on 9 January 2022).
